# Systems Genetics Reveals the Functional Context of PCOS Loci and Identifies Genetic and Molecular Mechanisms of Disease Heterogeneity

**DOI:** 10.1371/journal.pgen.1005455

**Published:** 2015-08-25

**Authors:** Michelle R. Jones, Meredith A. Brower, Ning Xu, Jinrui Cui, Emebet Mengesha, Yii-Der I. Chen, Kent D. Taylor, Ricardo Azziz, Mark O. Goodarzi

**Affiliations:** 1 Department of Medicine, Division of Endocrinology, Diabetes and Metabolism, Cedars-Sinai Medical Center, Los Angeles, California, United States of America; 2 Department Obstetrics and Gynecology, Division of Reproductive Endocrinology and Infertility, University of California Los Angeles, Los Angeles, California, United States of America; 3 The F. Widjaja Foundation Inflammatory Bowel and Immunobiology Research Institute, Cedars-Sinai Medical Center, Los Angeles, California, United States of America; 4 Institute for Translational Genomics and Population Sciences, Los Angeles Biomedical Research Institute at Harbor-UCLA Medical Center, Torrance, California, United States of America; 5 Departments of Obstetrics and Gynecology and Medicine, Medical College of Georgia, Georgia Regents University, Augusta, Georgia, United States of America; Pennsylvania State University College of Medicine, UNITED STATES

## Abstract

Genome wide association studies (GWAS) have revealed 11 independent risk loci for polycystic ovary syndrome (PCOS), a common disorder in young women characterized by androgen excess and oligomenorrhea. To put these risk loci and the single nucleotide polymorphisms (SNPs) therein into functional context, we measured DNA methylation and gene expression in subcutaneous adipose tissue biopsies to identify PCOS-specific alterations. Two genes from the *LHCGR* region, *STON1-GTF2A1L* and *LHCGR*, were overexpressed in PCOS. In analysis stratified by obesity, *LHCGR* was overexpressed only in non-obese PCOS women. Although not differentially expressed in the entire PCOS group, *INSR* was underexpressed in obese PCOS subjects only. Alterations in gene expression in the *LHCGR*, *RAB5B* and *INSR* regions suggest that SNPs in these loci may be functional and could affect gene expression directly or indirectly via epigenetic alterations. We identified reduced methylation in the *LHCGR* locus and increased methylation in the *INSR* locus, changes that are concordant with the altered gene expression profiles. Complex patterns of meQTL and eQTL were identified in these loci, suggesting that local genetic variation plays an important role in gene regulation. We propose that non-obese PCOS women possess significant alterations in LH receptor expression, which drives excess androgen secretion from the ovary. Alternatively, obese women with PCOS possess alterations in insulin receptor expression, with underexpression in metabolic tissues and overexpression in the ovary, resulting in peripheral insulin resistance and excess ovarian androgen production. These studies provide a genetic and molecular basis for the reported clinical heterogeneity of PCOS.

## Author Summary

Polycystic ovary syndrome (PCOS) is the most common hormonal disturbance in reproductive age women and features high levels of male sex hormones, such as testosterone, and infrequent ovulation. Twin studies have demonstrated that inheritance plays a significant role in PCOS, and recent genome wide association studies (GWAS) have implicated 11 susceptibility regions. The mechanism by which these genetic loci cause PCOS has yet to be determined. We looked at DNA methylation and gene expression levels in these 11 loci in fat biopsies from women with and without PCOS. We identified differences in the expression of two receptors that bind hormones known to contribute to the pathogenesis of PCOS–the receptors for luteinizing hormone (LH) and insulin. We found increased expression of the LH receptor in non-obese PCOS women, while in the obese women with PCOS the insulin receptor was underexpressed. Both excess LH stimulation and elevated insulin levels, due to decreased receptor levels and resulting insulin resistance, can cause increased androgen production from the ovary. Our findings suggest the primary mechanism for elevated androgen levels in PCOS may differ between non-obese and obese women with PCOS and that the clinical heterogeneity seen in PCOS may have genetic underpinnings.

## Introduction

Polycystic ovary syndrome (PCOS) occurs in 6–10% of reproductive age women by NIH diagnostic criteria, and is characterized by hyperandrogenism and oligo- or amenorrhea [1]. Metabolic risk factors for type 2 diabetes and cardiovascular disease such as insulin resistance and obesity are common in women with PCOS, with increased body weight, insulin resistance, and impaired glucose tolerance most elevated in women with the highest levels of androgens [1, 2].

PCOS is a complex disorder with both genetic and environmental factors contributing to its pathophysiology. Twin studies have provided heritability estimates for PCOS of 0.71 [3]. Two genome wide association studies (GWAS), carried out in Han Chinese populations, identified 15 risk SNPs from 11 loci (*THADA*, *LHCGR*, *FSHR*, *C9orf3*, *DENND1A*, *YAP1*, *RAB5B*, *INSR*, *TOX3*, *SUMO1P1*, and *HMGA2)* [4, 5]. Six of these risk loci (*THADA*, *LHCGR*, *FSHR*, *DENND1A*, *YAP1*, *INSR*) have been replicated in Caucasian populations [6–10]. In addition, a genetic risk score based on SNPs not individually associated with PCOS was found to be significantly associated with PCOS in Caucasian subjects [10], suggesting that some or all of the variants identified in Chinese populations are likely also risk variants in Caucasians.

GWAS have provided insight into the genetic architecture of many complex diseases, including PCOS. A limited number of functional studies have evaluated the role of several of the newly identified PCOS risk loci, including *LHCGR* (Luteinizing hormone/choriogonadotropin receptor) and *DENND1A* (DENN/MADD domain containing 1A) [11–13]. The *LHCGR* promoter region was shown to be hypomethylated and mRNA expression level increased in granulosa cells from women with PCOS [12]. An *in vitro* study reported overexpression of transcriptional variant 2 of *DENND1A* (*DENND1Av2*) in the theca cells of PCOS patients and demonstrated its ability to increase androgen and progestin biosynthesis [13]. These functional studies provide early evidence that alterations in methylation and gene expression within the PCOS GWAS susceptibility loci contribute to the pathophysiology of PCOS. In order to gain a greater understanding of the role of the PCOS susceptibility loci identified by GWAS further functional studies in PCOS relevant tissues are urgently needed.

The functional characteristics of a locus can include the epigenetic regulation of expression (for example, DNA methylation or histone modification), enhancer binding activity, transcription factor binding profiles, promoter activity, and the gene expression profile. DNA methylation plays an important role in the regulation of gene expression by affecting chromatin state and the ability of transcription factors, enhancers and insulators to bind DNA [14]. DNA methylation profiles are impacted by local SNPs, either directly by the creation/ablation of CpG residues, or indirectly [15], allowing SNPs in non-coding regions of the genome to have functional impacts on local gene regulation. Tissue specific methylation patterns contribute to gene expression profiles that delineate tissue function; therefore, SNPs may have tissue specific effects on disease pathways. Genetic variants that regulate methylation at CpG residues are known as methylation quantitative trait loci (meQTL). Genetic variation can also impact gene expression in a manner independent of methylation. Identification of genotype effects on gene expression level (expression quantitative trait loci, or eQTL) can help to identify the causal transcript in a disease-associated locus. Although each index SNP from the PCOS GWAS loci has been assigned to a gene, this was done following the common practice of selecting the nearest gene, without functional knowledge such as expression profiles of transcripts surrounding the index SNP.

In the present study, we measured DNA methylation and gene expression in adipose tissue of PCOS women and normal controls in order to better understand the functional elements surrounding PCOS-associated SNPs. As an endocrine tissue with a clear role in metabolic function and relative ease of collection, adipose tissue is highly suited for functional studies of PCOS genes, particularly those not directly related to androgen excess or ovarian function. Adipose dysfunction in PCOS has been widely reported; studies of subcutaneous adipocytes from PCOS women have demonstrated resistance to insulin stimulated glucose transport and inhibition of lipolysis [16, 17]. We have generated the first functional maps of PCOS loci, comparing methylation and gene expression patterns between PCOS patients and healthy controls and the interactions between the SNPs in these regions and local methylation (meQTL) and local gene expression (eQTL). The aim of our study was to use a systems biology approach to investigate patterns of gene regulation and expression in the genomic regions surrounding the previously identified PCOS susceptibility loci in a PCOS relevant tissue in order to understand the functional context of these loci.

## Results

### Subject demographics

PCOS subjects were not significantly older than controls, and no significant difference in BMI was detected. As expected, PCOS cases had elevated testosterone and hirsutism measured by modified Ferriman-Gallwey (mFG) score (Table 1).

**Table 1:** Baseline characteristics of PCOS and control subjects.

	PCOS (n = 23)	Controls (n = 13)	P value
Age (yr)	28 (26–30)	33 (27–40)	0.049
BMI (kg/m^2^)	30.9 (25.7–34.2)	25.5 (24–32.7)	0.24
Obesity (%)^a^	56.5	38.5	0.49
Total Testosterone (ng/dL)	32 (27–55.5)	28 (22.3–30.5)	0.075
**Free Testosterone (pg/dL)**	**4.9 (4–7.9)**	**2.3 (1.6–3.8)**	**0.002**
DHEAS (μg/dL)	236 (203–352.5)	199.5 (113.5–272.5)	0.064
**Fasting Glucose (mg/dL)**	**88.5 (83–91.8)**	**74.5 (68–91)**	**0.009**
Fasting Insulin (μIU/ml)	9 (5–15.3)	9.5 (3.5–11.5)	0.50
HOMA2-IR	0.99 (0.55–1.59)	1.09 (0.91–1.36)	0.88
**mFG**	**7 (3–10)**	**0 (0–1)**	**<0.0001**

Data presented as median (interquartile range). Values compared using Wilcoxon rank sum test. BMI, body mass index; DHEAS, dehydroepiandrosterone sulfate; HOMA2-IR, homeostasis model assessment of insulin resistance; mFG, modified Ferriman-Gallwey.

^a^ Values presented as percentage of total cases or controls. Values compared using Fisher’s exact test.

### Case/control analysis of gene expression and DNA methylation

**Fig 1:**
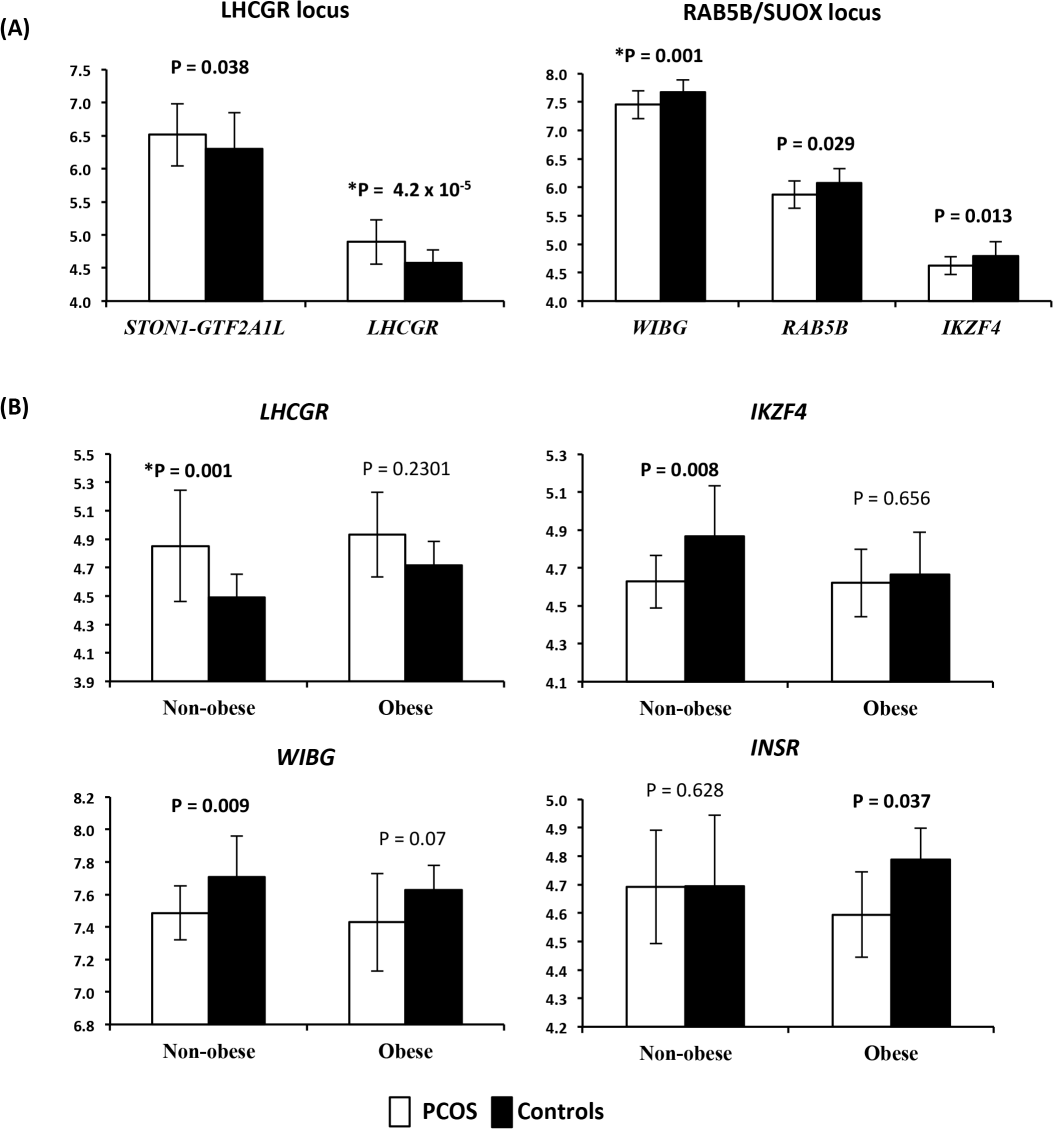
Expression levels of differentially expressed mRNA transcripts. **(A)** Expression levels of differentially expressed mRNA transcripts between PCOS and controls in the *LHCGR* and *RAB5B/SUOX* loci. On the X axis are gene names. On the Y axis are the mean expression levels. Error bars represent standard deviation. **(B)** Expression levels of mRNA transcripts differentially expressed between PCOS and controls, stratified by obesity. On the X axis is the obesity status of the subjects, non-obese and obese subjects analyzed separately. On the Y axis are the mean expression levels. * Denotes results that remained significant after correction for multiple testing. Error bars represent standard deviation.

In 35 subjects (22 cases and 13 controls), we examined the gene expression profiles for each transcript that passed normalization and background correction within the 11 PCOS risk loci. A total of 50 transcripts were identified in the genomic windows surrounding the PCOS risk variants and extracted from the genome wide expression dataset. Twenty-eight of these were expressed in the adipose tissue samples. Both *LHCGR* and *STON1-GTF2A1L* from the *LHCGR* locus were overexpressed in PCOS, while *WIBG*, *RAB5B* and *IKZF4* from the *RAB5B* locus were underexpressed in PCOS (Fig 1A). After correction for multiple testing with FDR, both *LHCGR* and *WIBG* remained significantly differentially expressed. Power estimates indicated we were well powered to detect significant effects at an alpha of 0.05 (power for detection of differences in expression of *LHCGR* was 0.93). In order to investigate the effect of obesity on gene expression in PCOS adipose tissue, we performed secondary analyses in obese and non-obese subjects separately (Fig 1B). In the non-obese subjects, *LHCGR* was significantly overexpressed in PCOS and *WIBG* and *IKZF4* were significantly underexpressed in PCOS. *INSR* was underexpressed only in obese PCOS subjects, with no changes in expression in non-obese PCOS women. *LHCGR* remained significantly overexpressed in non-obese subjects after correction for multiple testing; however, other stratified results were no longer significant (FDR P value <0.05).

**Fig 2:**
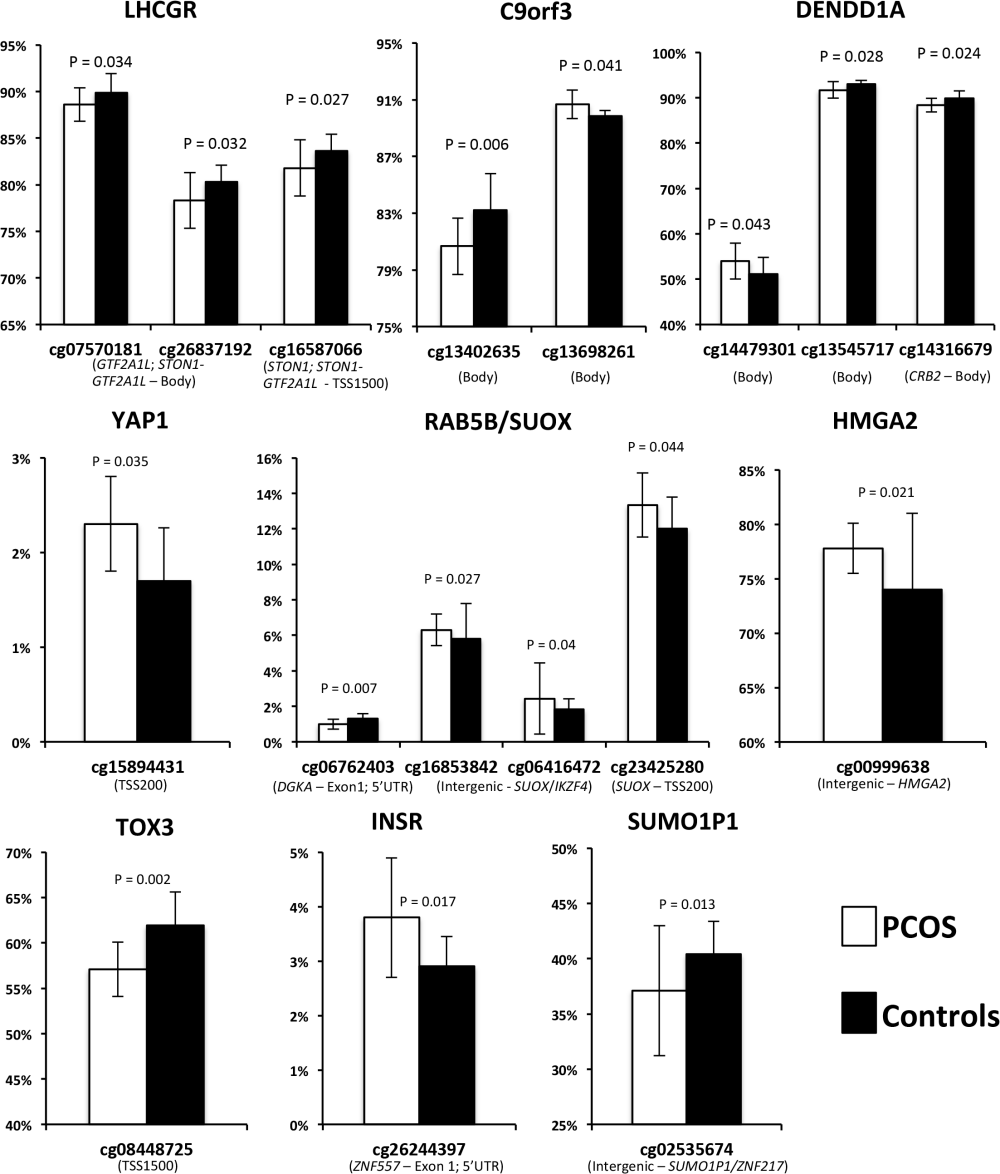
Methylation (Beta) levels at CpG sites with significantly different methylation between PCOS and controls. On the X axis are the significant CpG sites in the windows around the PCOS GWAS SNPs. On the Y axis is the methylation status, measured as the mean beta level. Error bars represent standard deviation.

Mean beta methylation level at a total of 650 CpG sites across the 11 PCOS risk loci windows were analyzed in 13 cases and 11 controls. A total of 17 CpG sites across the 11 windows demonstrated significant differences in methylation levels between PCOS subjects and controls (empirical P<0.05) (Fig 2). Four CpG sites were differentially methylated across the *RAB5B* window, including two sites located in the intergenic region 5’ to *IKZF4* with increased methylation in PCOS subjects (Fig 2). Within the *INSR* window a single CpG was hypermethylated in PCOS subjects. Three CpG sites in the *LHCGR* window, all located near *STON1-GTF2A1L*, were hypomethylated in PCOS subjects. CpG sites in the *C9orf3*, *DENND1A*, *YAP1*, *HMGA2*, *TOX3* and *SUMO1P1* loci were also differentially methylated between PCOS and controls (Fig 2). We applied FDR correction for multiple testing and did not identify any methylation sites that retained significance; however, due to the highly correlated nature of methylation probes we would consider this approach conservative. Correlation analysis between differentially methylated sites and expression level of genes within the local window did not reveal any significant expression quantitative methylation (eQTM).

Of the remaining eight loci, six had PCOS specific changes in methylation (Fig 2). We also examined each window for changes in gene expression in PCOS, however did not identify any other genes that are over/under-expressed in PCOS. Several windows contained genes that were not expressed in our adipose samples (S1 Table), including the DENND1Av2 transcriptional variant reported to be overexpressed in PCOS theca and urine [13]. We did identify reduced methylation in intron 2 of *DENND1A*, which may regulate isoform specific expression in a tissue dependent manner.

### Replication of differentially expressed genes in Gene Expression Omnibus (GEO)

The National Center for Biotechnology Information’s (NCBI) GEO database was used to further investigate the differentially expressed genes in our cohort both in subcutaneous adipose and other tissue types (S2 Table). In a small series comparing gene expression in subcutaneous adipose tissue of PCOS and control subjects, *WIBG* was underexpressed in PCOS patients, similar to our findings. Also consistent with our findings, in cumulus cells *LHCGR* was overexpressed in PCOS subjects. In the latter series, when the subjects were stratified by obesity, the non-obese PCOS subjects had lower expression of *WIBG* and *LHCGR* continued to be overexpressed. In the obese subjects, women with PCOS demonstrated higher expression of *INSR* in cumulus cells. Lower expression of *INSR* was seen in PCOS subjects in two different series of skeletal muscle.

### meQTL and eQTL

**Fig 3:**
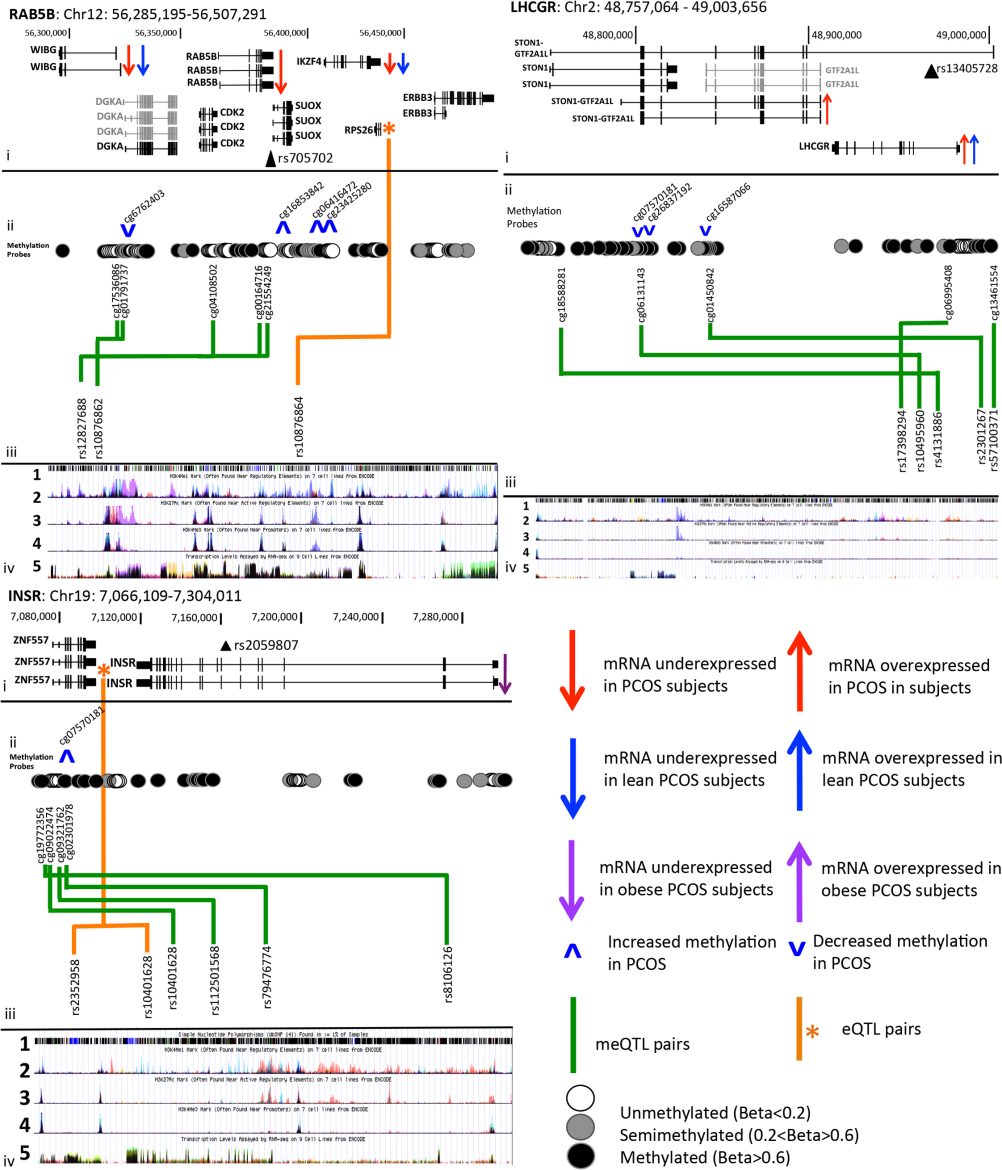
PCOS risk loci contain alterations in gene regulation and expression in PCOS adipose tissue. **i.** Chromosomal co-ordinates, gene structure and gene expression profile (grey = not expressed in adipose, black = expressed in adipose). The index PCOS GWAS risk SNP is marked by a filled black triangle and is labeled with rs number. **ii.** Methylation sites are shown as open (unmethylated), grey filled (semi-methylated) or black (fully methylated) circles, and meQTL relationships between these sites and local SNPs are shown with a green arrow. eQTL results are shown by an orange star marking the gene and orange arrows marking SNP position of independent signals. **iii.** UCSC Genome Browser ENCODE tracks show **1** SNP position from dbSNP143, **2** poised enhancer activity, **3** active enhancer activity, **4** active promoter activity and **5** transcriptional activity, in 7 Encode reference cell types. **iv.** meQTL results are shown with box and whisker plots demonstrating mean methylation (Beta level) in each genotype group.

Relationships between SNPs and methylation and gene expression were further investigated using a systems genetics approach. meQTL were identified in 19 subjects that had both methylation and genotype data available. Within the *LHCGR* window, SNPs in the 5’ and intron 1 regions of the *LHCGR* gene, surrounding the PCOS risk SNP rs13405728, were associated with methylation level of three CpG residues clustered in the *STON1-GTF2A1L* gene (S5 Table and Fig 3). Association of one of these methylation sites (cg01450842) with local variants has been previously reported in adipose tissue [14], suggesting that variants in the 5’ and intron 1 regions of *LHCGR* may play a role in methylation, and potentially transcriptional regulation of genes at this locus. The minor allele at each of these three meQTL pairings was associated with decreased methylation level at each site, suggesting these variants reduce methylation and may lead to increased expression (S5 Table and Figs 3 and S1).

Five SNPs (meQTLs) from across the *INSR* window were associated with 4 adjacent CpG sites clustered upstream and in intron 1 of *ZNF557* (S5 Table). The two most 5’ methylation sites in meQTL pairs (rs8106126-cg19772356, rs10401628-cg09022474) are upstream of the *ZNF557* gene and overlap enhancer, promoter and transcription factor binding sites in the ENCODE data track of Fig 3 (Panels iii-2,3,4). Within the gene body of *ZNF557* is a cluster of unmethylated probes, two of which were in meQTL pairs with SNPs in intron 11 and 12 of the INSR gene. One of these SNPs, rs8106125, is in moderate LD (r^2^ = 0.50) with the PCOS GWAS index SNP, shown in Fig 3 by a black triangle in track i, labeled as rs2059807. mRNA levels of *INSR* were associated with a number of SNPs across the 5’ region of the window (P values are shown in S4 Table) that are in a complex pattern of LD. A 4 SNP (rs10401628, rs2352958, rs7248939, and rs10418342) conditional regression analysis (shown in green in the regional association plot in S2 Fig) was required to remove any signal of significant association in the eQTL analysis results and suggests that at least 4 SNPs independently act as eQTLs in this window (Fig 3).

In the *RAB5B/SUOX* window four SNPs from across the window acted independently as meQTL SNPs with five methylation sites clustered between *WIBG* and *DGKA* and at the 5’ and 3’ of *RAB5B* (S5 Table). ENCODE data indicated that these methylation sites overlap active enhancer and promoter regions. Finally, an eQTL for this locus was also identified with many linked SNPs from across the window and *RPS26* (Fig 3). A conditional analysis with the top SNP (rs10876864) eliminated the significant associations from all other SNPs, indicating that a single association between many linked SNPs accounts for the numerous association signals.

## Discussion

To gain insight into the function of PCOS susceptibility loci we evaluated genotype, methylation and mRNA expression in the regions surrounding these SNPs in PCOS and healthy control adipose tissue. We have generated the first functional maps of PCOS GWAS loci in PCOS tissue, mapping methylation and gene expression surrounding previously identified PCOS risk loci and identifying relationships between genetic variants and these functional elements. These functional maps allowed us to identify PCOS specific changes in gene expression and methylation in several loci in PCOS adipose tissue. We have also identified differences in the gene expression profile of these risk genes in non-obese and obese PCOS subjects.

We found *LHCGR* was overexpressed in the adipose tissue of non-obese women with PCOS, and corresponding decreases in methylation of adjacent CpG residues. This is consistent with prior studies demonstrating increased *LHCGR* expression in granulosa and theca cells from patients with PCOS compared to normal controls [12]. We found this non-obese specific increase in expression was also present in cumulus cells from women with PCOS in our confirmatory analysis from the GEO database (S2 Table). Women with PCOS, particularly when not obese, have higher levels of LH secreted from the pituitary [18–20], increased bioactivity of LH [21, 22] and excessive production of androgens from the ovaries in response to LH [18, 23, 24]. It is possible that enhanced sensitivity to LH in the ovary is due to increased receptor number as a result of overexpression of *LHCGR*, resulting in elevated androgen synthesis from the theca cell.

A biological role for *LHCGR* in adipose is not clear. The Genotype-Tissue Expression Project (GTEx) database [25] reports its expression in subcutaneous adipose as well as several other unexpected tissues such as visceral adipose (omentum), tibial nerve, and esophagus. Reduced methylation and overexpression of *LHCGR* in adipose could represent a conserved gene regulation profile across tissues in non-obese women with PCOS. To confirm that our finding of *LHCGR* overexpression is a PCOS-specific effect, we identified GEO datasets that could be analyzed with either obesity or insulin sensitivity as a dichotomous trait. We did not find any changes in expression between lean and obese subjects in three adipose GEO datasets where obesity was available to stratify subjects (S3 Table), or in three datasets where insulin sensitivity was available as a dichotomous trait. These findings, together with our own results, suggest that the observed changes in *LHCGR* expression are private to PCOS, and not a result of metabolic heterogeneity in the cohort.

The role of insulin in PCOS has been widely studied [26]. While insulin resistance is a common feature in PCOS women, it is particularly common in obese women with PCOS [27, 28]. Compensatory increased circulating insulin levels contribute to PCOS by stimulating ovarian androgen production and inhibiting hepatic SHBG production [29, 30]. In our study of adipose tissue, we found that obese women with PCOS had significantly lower expression of *INSR*. In keeping with this, *INSR* was also down regulated in skeletal muscle of PCOS patients in two independent studies (S2 Table). Decreased *INSR* expression in metabolic tissues is consistent with insulin resistance and provides a potential mechanism for insulin resistance frequently seen in obese women with PCOS.

Contrary to decreased *INSR* expression in metabolic tissues (adipose and skeletal muscle) of obese PCOS women, we found *INSR* to be overexpressed in the cumulus cells of obese PCOS subjects (S2 Table). Studies have demonstrated differences in insulin sensitivity between reproductive and metabolic tissues, where obese mice had a blunted response to insulin in the liver and muscle while the pituitary and ovary maintained insulin sensitivity [31]. Studies in insulin-resistant PCOS women suggest that the ovaries remain sensitive to insulin’s actions on steroidogenesis, even when metabolic tissues demonstrate peripheral insulin resistance by decreased glucose disposal [30]. Our finding of tissue specific underexpression of *INSR* in metabolic tissues and overexpression in ovarian tissues supports the previously suggested hypothesis of selective insulin resistance in PCOS, where ovarian sensitivity to insulin is maintained despite peripheral insulin resistance, allowing insulin driven androgen synthesis in the ovary to persist. We identified increased methylation of a single CpG site in a largely unmethylated region 5’ to the *INSR* transcription start site that also overlaps a regulatory motif in the UCSC ENCODE browser that could regulate *INSR* expression. Future experiments should include mapping the methylation and expression profile of *INSR* from several PCOS ovarian cell types, potentially supporting the hypothesis of maintained insulin sensitivity in the ovary as a result of alterations in *INSR* methylation and expression.

It is known that obese women with PCOS have significantly more insulin resistance and the LH levels are higher in non-obese women with PCOS [32]. Our findings suggest that the mechanisms underlying hyperandrogenemia in obese and non-obese PCOS may have a different genetic basis. Non-obese women with increased *LHCGR* expression may have increased LH-dependent androgen production by the ovary due to increased number of LH receptors and increased LH levels. Obese women with increased *INSR* expression in androgen-synthesizing ovarian cells may have hyperandrogenemia driven by the hyperinsulinemic response to reduced insulin receptor number in metabolic tissues.

We also identified a number of changes in gene regulation and expression in the *RAB5B* window. In PCOS samples *WIBG* was underexpressed at FDR corrected significance, and reduced expression levels of *RAB5B*, and *IKZF4* were nominally associated with PCOS. Increased methylation was observed at three CpG sites across the locus, but did not meet correction for multiple testing. Our restricted sample size in methylation analysis, and in stratified expression analysis likely reduced our ability to detect smaller effects. While expression and methylation levels were not significantly correlated in an eQTM relationship, we assayed a relatively small number of all potential methylation sites from this locus, and more extensive changes in methylation at unassayed residues may be regulated by eQTM for these genes. We measured methylation in a subset (24 of our total 36 samples) of adipose samples, and while this is the largest study of this type published to date, the relatively small sample size may have reduced our ability to identify eQTM. A publicly available replication data set comparing gene expression between PCOS and controls in subcutaneous adipose tissue also found *WIBG* to be underexpressed in PCOS (S2 Table). *WIBG* encodes a cytoplasmic protein that binds to the ribosomal unit and increases translational efficiency of mRNA [33, 34]. A specific role for *WIBG* in PCOS is unclear.

*RAB5B* is a small GTPase that plays a role in early endosome formation and is required for the endocytic pathway that mediates the transport of clathrin-coated vesicles from the plasma membrane to the early endosome [35]. *RAB5B* has also been identified as a susceptibility locus for type 1 diabetes and childhood obesity [36]. Interestingly, *DENND1A* encodes for a protein, connecdenn 1, that also facilitates endocytosis and membrane trafficking and is known to interact with Rab family member *RAB35* [37]. Functional studies of *DENND1A* demonstrated increased expression of *DENND1Av2* and increased androgen synthesis in the theca cells of PCOS women [13]. This variant was not expressed in our adipose samples. Given *RAB5B’s* association with type 1 diabetes it is possible that genes in this locus play a regulatory role via that impacts beta cell function or insulin secretion, a process that is impaired in both disorders.

A third gene, *IKZF4*, was also down regulated in subcutaneous adipose of women with PCOS. *IKZF4* is zinc-finger transcription factor that functions as a transcriptional repressor and is known to play a role in immune regulation, specifically in the programming of T regulatory cells [38]. There is evidence suggesting the presence of chronic low-grade inflammation in women with PCOS; studies have found significantly higher levels of C-reactive protein (CRP) and other cytokines, independent of BMI [39]. Underexpression of *IKZF4* in PCOS adipose tissues may impact the ability of T cells to suppress pro-inflammatory responses, and contribute to the chronic inflammation seen in PCOS. As several markers of inflammation have been correlated with insulin resistance [40–42], chronic low-grade inflammation may contribute to the etiology of insulin resistance seen in PCOS.

In conclusion, PCOS GWAS loci contain extensive alterations in methylation and gene expression profiles between PCOS and controls, which identify genetic and molecular differences between clinical disease subtypes based on presence or absence of obesity. We demonstrated that LHCGR is overexpressed in the subcutaneous adipose tissue of non-obese PCOS women and *INSR* was underexpressed in obese women with PCOS. This underexpression of *INSR* in obese women with PCOS was also seen in cumulus cells. Taken together, our findings suggest that the gene expression profiles may be different between obese and non-obese PCOS subjects, with hormonal disturbances playing a more important role in non-obese subjects and metabolic disturbances playing a larger role in obese subjects. Our results suggesting different mechanisms underlying hyperandrogenemia in non-obese versus obese women may one day have clinical implications, as subclassification based on pathophysiology may lead to tailored treatment. While we did not resolve all functional regulatory mechanisms in PCOS loci in adipose tissue, we provide new insight into several of the susceptibility loci discovered in the PCOS GWAS. Given that methylation and expression vary between tissue types, further studies in other tissues relevant to PCOS pathophysiology are needed to further elucidate the function of these PCOS susceptibility loci.

## Materials and Methods

### Ethics statement

This study was approved by the Cedars-Sinai Institutional Review Board (IRB) under approval number 11289. All subjects gave written informed consent according to the guidelines of the IRB

### Study subjects

Subcutaneous lower abdominal adipose tissue was obtained from 23 PCOS and 13 control subjects using a previously described protocol for acquiring and processing subcutaneous adipose tissue [43]. PCOS subjects were recruited at a tertiary care academic institution. Cases were premenopausal, nonpregnant, and on no hormonal therapy, including oral contraceptives, for at least 3 months, and met 1990 National Institutes of Health criteria for PCOS [44]. Parameters for defining hirsutism, hyperandrogenemia, ovulatory dysfunction, and exclusion of related disorders were previously reported [45]. Controls were recruited by word of mouth and advertisements to the public calling for healthy women. Controls were healthy women, with regular menstrual cycles and no evidence of hirsutism, acne, alopecia, or endocrine dysfunction and had not taken hormonal therapy (including oral contraceptives) for at least 3 months. Clinical characteristics for these subjects are shown in Table 1.

### DNA/RNA extraction

Samples were snap frozen immediately after collection in liquid nitrogen and then stored at -80°C until extraction. DNA and RNA were isolated from subcutaneous fat tissue after rapid thaw at 37°C with the AllPrep DNA/RNA/protein Mini kit (QIAGEN, Valencia, CA). DNA was stored in TE buffer, quantified and checked for quality on a Nanodrop-1000 (Nanodrop, Wilmington, DE) and stored at -80°C. RNA samples were quantified and checked for quality using the BioAnalyzer 6000 Pico kit (Agilent, Santa Clara, CA) and stored at -80°C.

### Genotyping

Genotyping of 36 samples was performed at CSMC using the HumanExome chip (targeting functional (e.g., missense and splice junction) variants), the HumanOmniExpress chip (targeting common variants using a haplotype tagging approach) and the HumanOmni1S chip (targeting rare and non-Caucasian SNPs and copy number variants) following the manufacturer’s protocol (Illumina, San Diego, CA) [46, 47]. Samples were randomized by case/control status and arrayed at a concentration of 50ng/ul prior to genotyping as part of larger experiments. Thirty one samples passed sample based quality control measures for all three chips that included genotyping rate >98% (five samples had a genotyping rate <98%), p10GC (a sample statistic representing the tenth percentile of the distribution of genotype quality scores across all SNPs genotyped) and SNP-based gender estimate (all samples passed gender estimation). Genotypes from each chip were exported from Genome Studio (Illumina, San Diego, CA) and merged in SVS (Golden Helix, Bozeman, MT). SNPs with MAF>5% and Hardy-Weinberg Equilibrium P Value >1.0x10^-4^ were retained for downstream analysis (total number of SNPs carried forward was 1,180,811). Principal components analysis (PCA) within SVS was used to generate the top 10 PCs to identify outlier samples, of which none were found.

### Methylation

A subset of 24 age and BMI matched subjects were selected for methylation analysis due to restrictions on sample size because samples were run as part of a larger project. DNA methylation levels were measured using the HumanMethylation450 chip (Illumina, San Diego, CA) according to the manufacturer’s instructions at CSMC. The HumanMethylation450 chip targets over 485,000 CpG residues across 96% of all RefSeq genes (21,500 gene symbols) and 95% of CpG islands and flanking regions. Samples were randomized by case/control status across two plates (10 chips) in the context of a larger experiment and arrayed at a concentration of 10ng/ul. Detection P values were calculated to identify failed probes, and beta (β) values representing methylation levels were generated from the Genome Studio software for each methylation site, ranging from 0 (completely unmethylated) to 100% (completely methylated). The Methylumi package in R was used to background normalize and log transform the beta values [48]. 485,577 probes were exported from Methylumi for further analysis. The data was checked for distribution of the mean β value per site, distribution of the mean β value per site in each bead type, mean methylation score across all samples per CpG location, variance of the β levels across individuals, PC analysis and plotting for each sample and the distribution of methylation sites based on location relative to each CpG locus. All 24 samples passed QC measures and were retained for downstream analysis. Methylation level at individual probes was categorized as low-methylated (beta <0.4), semi-methylated (beta 0.4–0.6), or highly-methylated (beta >0.6).

### Gene expression

The HumanHT-12v4 beadchip was used to measure gene expression levels of well-characterized genes, gene candidates, and splice variants with 47,000 probes at the UCLA Neuroscience Genomics Core (UNGC). All 36 samples were randomized according to case/control status and RNA was arrayed at 10ng/ul. The TargetAmp-Nano Labeling Kit for Illumina Expression BeadChip (Epicenter, San Diego, CA) was used to label samples. Sample probe profile data was exported from Genome Studio after QC metrics (direct hyb control metrics including hybridization controls, stringency metrics, background and noise of control probes, gene intensity of housekeeping and all genes and labeling and background metrics) and sample metrics (number of genes detected, 95^th^ intensity percentile, signal to noise ratio, signal across all samples) were reviewed. One sample was excluded due to excessive signal to noise ratio. Sample probe profile data was read into the limma package in R version 3.1.1 [49]. Probes expressed in at least three samples and with a detection P value of <0.05 were retained (47,314 probes were read in and 29,081 probes were retained after this step). Background normalization, quantile normalization and log transformation of the remaining probes was performed with the neqc() function in limma. Normalized and transformed data was then used to generate PCs using the MDS() function within limma and PCs were plotted with samples labeled with case/control status to identify QC outliers for removal. No samples were outliers or flagged for removal at this step. Normalized and transformed data was exported from limma for analysis. An experimental flowchart describing sample size and data available for DNA genotyping, methylation and gene expression analysis is shown in S3 Fig.

### Definition of PCOS loci genomic windows

Our analysis was focused on genomic windows around each of the 11 loci previously discovered to harbor SNPs associated with PCOS in GWAS. The genomic region 100kb upstream and downstream of each GWAS SNP was evaluated for methylation and mRNA expression. If the window terminated within the coding frame of a gene, the window was extended to 10kb beyond the coding frame (S1 Table).

### Statistical analysis

Normalized and log transformed methylation beta levels and gene expression levels were analyzed in case/control analysis using logistic regression in SVS adjusting for age and BMI. Subjects were stratified by obesity status where subjects with BMI ≥ 30kg/m^2^ were categorized as obese, and subjects with BMI < 30kg/m^2^ were categorized as non-obese. Obesity stratified analyses were adjusted for age only. Linear regression in SVS adjusting for age, BMI, disease status and PC1 was used for meQTL (SNP associated with methylation level), eQTL (SNP associated with mRNA level) and eQTM (methylation level associated with mRNA level) analysis. meQTL relationships between methylation probes and multiple SNPs were interrogated for linkage disequilibrium (LD) between the SNPs, and conditional analysis using additive model genotype as a covariate in the linear regression was used to identify the variant driving the meQTL association if possible. We applied correction for multiple testing in gene expression and methylation results using the False Discovery Rate [50], with an FDR P value <0.05 held as significant. In light of the relatively small number of independent tests being run within each independent locus, we considered results with an empirical P value of <0.05 suggestive of significance. Correction for multiple testing for meQTL, eQTL and eQTM analysis was calculated on a per-window basis to adjust for the number of SNPs analyzed in a modified Bonferroni approach.

### Replication in Gene Expression Omnibus (GEO)

NCBI’s GEO is a public repository that archives high-throughput functional genomics data. To compare expression levels of the candidate genes discovered in our cohort in additional PCOS tissues we evaluated these genes in other datasets. A search of the GEO database identified 8 datasets comparing gene expression between PCOS patients and controls in various tissue types (S2 Table). The GEO2R interactive web tool was used to perform comparisons between PCOS and control subjects on the original submitter-supplied processed data tables. GEO2R uses GEOquery and limma R packages from the Bioconductor project to perform statistical analysis [51]. All data was log transformed. These analyses were not adjusted for age or BMI, as these traits are not available in the GEO database.

### Regulatory element look-up in Encyclopedia of Regulatory Elements (ENCODE)

ENCODE tracks were displayed in the UCSC Genome Browser using Build37 (GRCh37/hg19) in order to identify poised enhancer (H3K4Me1), active enhancer (H3K27Ac), active promoter (H3K4Me3), and transcription activity in the ENCODE reference cell types [52].

## Supporting Information

S1 Table Definition of the genomic windows around the SNPs previously associated with PCOS in GWAS. (DOCX)

S2 Table Analysis of differentially expressed genes in PCOS adipose (*LHCGR*, *WIBG*, *RAB5B*, *IKZF4*, *INSR*) in 7 datasets from NCBI’s Gene Expression Omnibus (GEO) repository. (DOCX)

S3 Table Analysis of *LHCGR* expression in 9 GEO datasets show changes in *LHCGR* expression are not due to obesity or changes in insulin sensitivity. (DOCX)

S4 Table Results of eQTL analysis passing correction for multiple testing for each PCOS locus. (XLSX)

S5 Table Results of meQTL analysis passing correction for multiple testing for each PCOS locus. (XLSX)

S1 Fig Associations between genotype and methylation levels for meQTL pairings in the *RAB5B*, *INSR* and *LHCGR* genomic loci. Genomic co-ordinates and gene position are shown in the top of the panel with the location of the PCOS GWAS index SNP shown as a solid triangle. Methylation status of each CpG residue is demonstrated by a circle (open = low-methylated, grey = semi-methylated, black = highly-methylated), with CpG-SNP interactions shown by a black line connecting associated methylation probes and SNPs. Box and whisker plots below show methylation level by genotype for each meQTL.
(TIFF)

S2 Fig Associations between genotype and mRNA expression levels for eQTL pairing in the *RAB5B* and *INSR* genomic loci. Genomic co-ordinates and gene position are shown in the top of the panel with the location of the PCOS GWAS index SNP shown as a solid triangle. Methylation status of each CpG residue is demonstrated by a circle (open = low-methylated, grey = semi-methylated, black = highly-methylated), with CpG-SNP interactions shown by a black line connecting associated methylation probes and SNPs. Box and whisker plots below show methylation level by genotype for each meQTL.
(TIFF)

S3 Fig Experiment flowchart. The samples used in this this study and the datasets generated for each.
(TIF)
