# Supplementary material for: Systems Genetics Reveals the Functional Context of PCOS Loci and Identifies Genetic and Molecular Mechanisms of Disease Heterogeneity
Source: PLoS Genet. 2015 Aug 25;11(8):e1005455. doi: 10.1371/journal.pgen.1005455 (PMC4549292; doi:10.1371/journal.pgen.1005455)
Supplement: S1 Table — (DOCX) [file pgen.1005455.s001.docx]

|  | | | | | | | |  |
| --- | --- | --- | --- | --- | --- | --- | --- | --- |
|  |  |  |  |  |  |  |  |  |
| **Gene** | **Independent GWAS SNP(s)** | **Chromosome** | **Window Coordinates***  **(size of window)** | **# of CpG sites** | **# SNPs** | **# mRNA transcripts** | **Transcripts in window**** |  |
|  |  |  |  |  |  |  |  |  |
| THADA | rs13429458 | 2 | 43,413,274-43,864,401 (451,127 bp) | 82 | 158 | 4 | **THADA (ILMN_1706818) THADA (ILMN_1811624) ZFP36L2 (ILMN_2150258) LOC100129726 (ILMN_3187435)** |  |
|  | rs12478601 |  |  |  |  |  |  |  |
|  |  |  |  |  |  |  |  |  |
| LHCGR | rs13405728 | 2 | 48,747,064-49,078,159 (331,095 bp) | 56 | 233 | 6 | **LHCGR (ILMN 1788098) STON1_GTF2A1L (ILMN 1656409) GTF2A1L (ILMN 1681616) GTF2A1L (ILMN 1716041**) GTF2A1L (ILMN 1682943)  STON1 (ILMN1668592 ) |  |
|  |  |  |  |  |  |  |  |  |
|  |  |  |  |  |  |  |  |  |
| FSHR | rs2268361 | 2 | 49,101,362-49,391,666 (290,304 bp) | 10 | 203 | 2 | FSHR (ILMN_1661616) FSHR (ILMN_2333449) |  |
|  | rs2349415 |  |  |  |  |  |  |  |
| C9orf3 | rs3802457 | 9 | 97,478,951-97,859,500 (380,549 bp) | 53 | 65 | 5 | C9orf3 (ILMN_1674629) **MIR2278 (ILMN_3309294)** MIR23B (ILMN_3308798) MIR27B (ILMN_3310356) MIR24-1 (ILMN_3310401) |  |
|  | rs4385527 |  |  |  |  |  |  |  |
|  |  |  |  |  |  |  |  |  |
| DENND1A | rs10818854 | 9 | 126,108,448-126,702,417 (593,969 bp) | 54 | 219 | 5 | **DENND1A (ILMN_1753275)** DENND1A (ILMN_1727315) DENND1A (ILMN_1728073) CRB2 (ILMN_1681826) MIR601 (ILMN_3310518) |  |
|  | rs2479106 |  |  |  |  |  |  |  |
|  |  |  |  |  |  |  |  |  |
| YAP1 | rs1894116 | 11 | 101,970,639-102,171,639 (201,000 bp) | 38 | 52 | 1 | **YAP1 (ILMN_1709479)** |  |
| RAB5B/SUOX | rs705702 | 12 | 56,285,195-56,507,291 (222,096 bp) | 165 | 39 | 18 | **RAB5B (ILMN_1752582) SUOX (ILMN_1803745) SUOX (ILMN_1710682) SUOX (ILMN_2383455) IKZF4 (ILMN_1741334)** RPS26 (ILMN_1755664) **RPS26 (ILMN_2209027) ERBB3 (ILMN_1737993) ERBB3 (ILMN_2397602)** **ERBB3 (ILMN_1751346) CDK2 (ILMN_1653443) CDK2 (ILMN_1665559)** DGKA (ILMN_1661454) DGKA (ILMN_1747132) **DGKA (ILMN_2319910)** DGKA (ILMN_1661544) DGKA (ILMN_2319913) **WIBG (ILMN_1751431)** |  |
|  |  |  |  |  |  |  |  |  |
|  |  |  |  |  |  |  |  |  |
|  |  |  |  |  |  |  |  |  |
|  |  |  |  |  |  |  |  |  |
|  |  |  |  |  |  |  |  |  |
|  |  |  |  |  |  |  |  |  |
|  |  |  |  |  |  |  |  |  |
| HMGA2 | rs2272046 | 12 | 66,124,461-66,370,071 (245,610 bp) | 60 | 72 | 3 | **HMGA2 (ILMN_1666236)**  HMGA2 (ILMN_1671547)  HMGA2 (ILMN_2344662) |  |
| TOX3 | rs4784165 | 16 | 52,247,819-52,590,806 (342,987 bp) | 29 | 133 | 27 | TOX3 (ILMN_2413833) MIR548A1 (ILMN_3310683)  MIR548A2 (ILMN_3309259) MIR548A3 (ILMN_3310020) MIR548B (ILMN_3309980) MIR548C (ILMN_3309006) MIR548E (ILMN_3308350) MIR548F1 (ILMN_3310990) MIR548F2 (ILMN_3309794)  MIR548F3 (ILMN_3310241)  MIR548F4 (ILMN_3310226) MIR548F5 (ILMN_3310945) MIR548G (ILMN_3310553)  MIR548H1 (ILMN_3309565) MIR548H3 (ILMN_3308668) MIR548H4 (ILMN_3308250) MIR548I1 (ILMN_3308540) MIR548I2 (ILMN_3308843)  MIR548I4 (ILMN_3308285) MIR548J (ILMN_3308020) **MIR548K (ILMN_3308495)**  MIR548L (ILMN_3308450) MIR548M (ILMN_3308300) MIR548N (ILMN_3309519) MIR548O (ILMN_3310266) MIR548P (ILMN_3311120)  MIR548Q (ILMN_3310578) |  |
|  |  |  |  |  |  |  |  |  |
| INSR | rs2059807 | 19 | 7,059,271-7,304,011  (244,740 bp) | 67 | 207 | 3 | **INSR (ILMN_1670918) ZNF557 (ILMN_2346137) ZNF557 (ILMN_1719163)** |  |
|  |  |  |  |  |  |  |  |  |
| SUMO1P1 | rs6022786 | 20 | 52,173,610-52,547,303 (373,693 bp) | 36 | 225 | 3 | SUMO1P1 (2195390) SUMO1P1 (1785615) **ZNF217 (1755303)** |  |

| * Position in Genome Reference Consortium Human Build 37 |  |  | |  |  |  |  | |  | |  |
| --- | --- | --- | --- | --- | --- | --- | --- | --- | --- | --- | --- |
| ** Genes listed as gene name (Illumina ID probe). Bold genes passed normalization and are expressed in adipose tissue. | | | | |  |  |  | |  | |  |
|  | | | | | | | | | | |  |
|  |  | |  |  |  |  | |  | |  |  |
