# Supplementary material for: Systems Genetics Reveals the Functional Context of PCOS Loci and Identifies Genetic and Molecular Mechanisms of Disease Heterogeneity
Source: PLoS Genet. 2015 Aug 25;11(8):e1005455. doi: 10.1371/journal.pgen.1005455 (PMC4549292; doi:10.1371/journal.pgen.1005455)
Supplement: S2 Table — (DOCX) [file pgen.1005455.s002.docx]

| **PubMed**  **ID** | **GEO ID** | **Probe_ID** | **Gene** | **Fold Change** | **Regulation** | **P Value** | **Samples Description** | **Platform** | |
| --- | --- | --- | --- | --- | --- | --- | --- | --- | --- |
| 19141487 | GSE10946 | 207240_s_at | LHCGR | 2.11 | UP | **0.0078** | Cumulus cells  12 PCOS and11 controls | Affymetrix  HG-U133_Plus_2 | |
| 19141487 | GSE10946 | 207240_s_at | LHCGR | 3.06 | UP | **0.0068** | Cumulus cells  **Lean**: 5 PCOS and 6 controls | Affymetrix  HG-U133_Plus_2 | |
| 19141487 | GSE10946 | 207240_s_at | LHCGR | 1.53 | UP | 0.25 | Cumulus cells  **Obese**: 7 PCOS and 5 controls | Affymetrix  HG-U133_Plus_2 | |
| 19141487 | GSE10946 | 242304_at | WIBG | 1.02 | UP | 0.91 | Cumulus cells  12 PCOS and 11 controls | Affymetrix  HG-U133_Plus_2 | |
| 19141487 | GSE10946 | 242304_at | WIBG | 1.41 | UP | **0.031** | Cumulus cells  **Lean**: 5 PCOS and 6 controls | Affymetrix  HG-U133_Plus_2 | |
| 19141487 | GSE10946 | 242304_at | WIBG | 0.73 | DOWN | 0.17 | Cumulus cells  **Obese**: 7 PCOS and 5 controls | Affymetrix  HG-U133_Plus_2 | |
| None | GSE43264 | 84305_at | WIBG | 0.60 | DOWN | **0.0025** | Sub-cutaneous adipose tissue  8 PCOS and 7 controls  Age- and BMI matched | Affymetrix NuGO_Hs1a520180 | |
| 22904171 | GSE34526 | 201276_at | RAB5B | 2.94 | UP | **0.0078** | Granulosa cells  7 PCOS subjects and 3 controls | Affymetrix  HG-U133_Plus_2 | |
| 23824412 | GSE48301 | 7956088 | RAB5B | 1.40 | UP | **0.0077** | Endometrial stromal fibroblasts  **Overweight/obese**: 4 PCOS and 4 controls | Affymetrix  HuGene-1_0-st | |
| 23824412 | GSE48301 | 7956105 | IKZF4 | 1.43 | UP | **0.0301** | Endometrial endothelial cells  **Overweight/obese**: 3 PCOS and 4 controls | Affymetrix  HuGene-1_0-st | |
| 17563058 | GSE6798 | 226450_at | INSR | 0.82 | DOWN | **6.95 x 10^-6^** | Skeletal muscle  **Obese**: 16 PCOS and 13 controls | Affymetrix  HG-U133_Plus_2 | |
| 18560589 | GSE8157 | 226450_at | INSR | 0.83 | DOWN | **5.85 x 10^-4^** | Skeletal muscle  **Obese**:10 PCOS and 13 controls | Affymetrix  HG-U133_Plus_2 | |
| 19141487 | GSE10946 | 226216_at | INSR | 1.63 | UP | 0.086 | Cumulus cells  12 PCOS and11 controls | Affymetrix  HG-U133_Plus_2 | |
| 19141487 | GSE10946 | 226216_at | INSR | 0.93 | DOWN | 0.86 | Cumulus cells  **Lean**: 5 PCOS and 6 controls | Affymetrix  HG-U133_Plus_2 | |
| 19141487 | GSE10946 | 226216_at | INSR | 2.55 | UP | **0.0063** | Cumulus cells  **Obese**: 7 PCOS and 5 controls | Affymetrix  HG-U133_Plus_2 | |
| 23824412 | GSE48301 | 8033362 | INSR | 1.43 | DOWN | **0.0024** | Endometrial endothelial cells  **Overweight/obese:** 3 PCOS and 4 controls | Affymetrix  HuGene-1_0-st | |
| 17062763 | GSE5090 | LHCGR, WIBG, RAB5B, IKZF4 and INSR were not differentially expressed between PCOS and control subjects | | | | | Omental adipose tissue  **Obese**: 8 PCOS and 7 controls | Affymetrix  HG-U133A |  |
| 17148555 | GSE5850 | LHCGR, WIBG, RAB5B, IKZF4 and INSR were not differentially expressed between PCOS and control subjects | | | | | MII oocytes  6 PCOS and 6 controls | Affymetrix  HG-U133_Plus_2 |  |
