# Supplementary material for: Systems Genetics Reveals the Functional Context of PCOS Loci and Identifies Genetic and Molecular Mechanisms of Disease Heterogeneity
Source: PLoS Genet. 2015 Aug 25;11(8):e1005455. doi: 10.1371/journal.pgen.1005455 (PMC4549292; doi:10.1371/journal.pgen.1005455)
Supplement: S3 Table — (DOCX) [file pgen.1005455.s003.docx]

| **GEO ID** | **Gene** | **Probe_ID** | **P Value** | **Samples Description** | **Platform** |
| --- | --- | --- | --- | --- | --- |
| GSE48964 | LHCGR | 8052058 | 0.49 | Subcutaneous adipose from 3 obese (BMI>40kg/m^2^) and 3 lean (BMI<25kg/m^2^), men and women. | Affymetrix  Human Gene 1.0STArray |
| GSE29718 | LHCGR | 8052058 | 0.96 | Subcutaneous adipose from 2 obese women and 4 lean women. | Affymetrix  Human Gene 1.0STArray |
| GSE2510 | LHCGR | 207240_s_at | 0.90 | Cultured abdominal subcutaneous preadipocytes from 7 lean and 7 obese women. | Affymetrix Human Genome U133A Array |
| GSE15773 | LHCGR | 207240_s_at | 0.75 | Subcutaneous adipose from 4 obese insulin resistant subjects and 5 obese insulin sensitive subjects, men and women included. | Affymetrix Human Genome U133 Plus 2.0 Array |
| GSE20950 | LHCGR | 207240_s_at | 0.30 | Subcutaneous adipose from 5 obese insulin resistant women and 8 obese insulin sensitive women. | Affymetrix Human Genome U133 Plus 2.0 Array |
| GSE62832 | LHCGR | 8052058 | 0.24 | Subcutaneous adipose from 11 metabolically normal subjects and 7 metabolically abnormal subjects, men and women included. | Affymetrix Human Gene 1.0 ST Array |
