# Supplementary figures and images for: Systems Genetics Reveals the Functional Context of PCOS Loci and Identifies Genetic and Molecular Mechanisms of Disease Heterogeneity
Source: PLoS Genet. 2015 Aug 25;11(8):e1005455. doi: 10.1371/journal.pgen.1005455 (PMC4549292; doi:10.1371/journal.pgen.1005455)

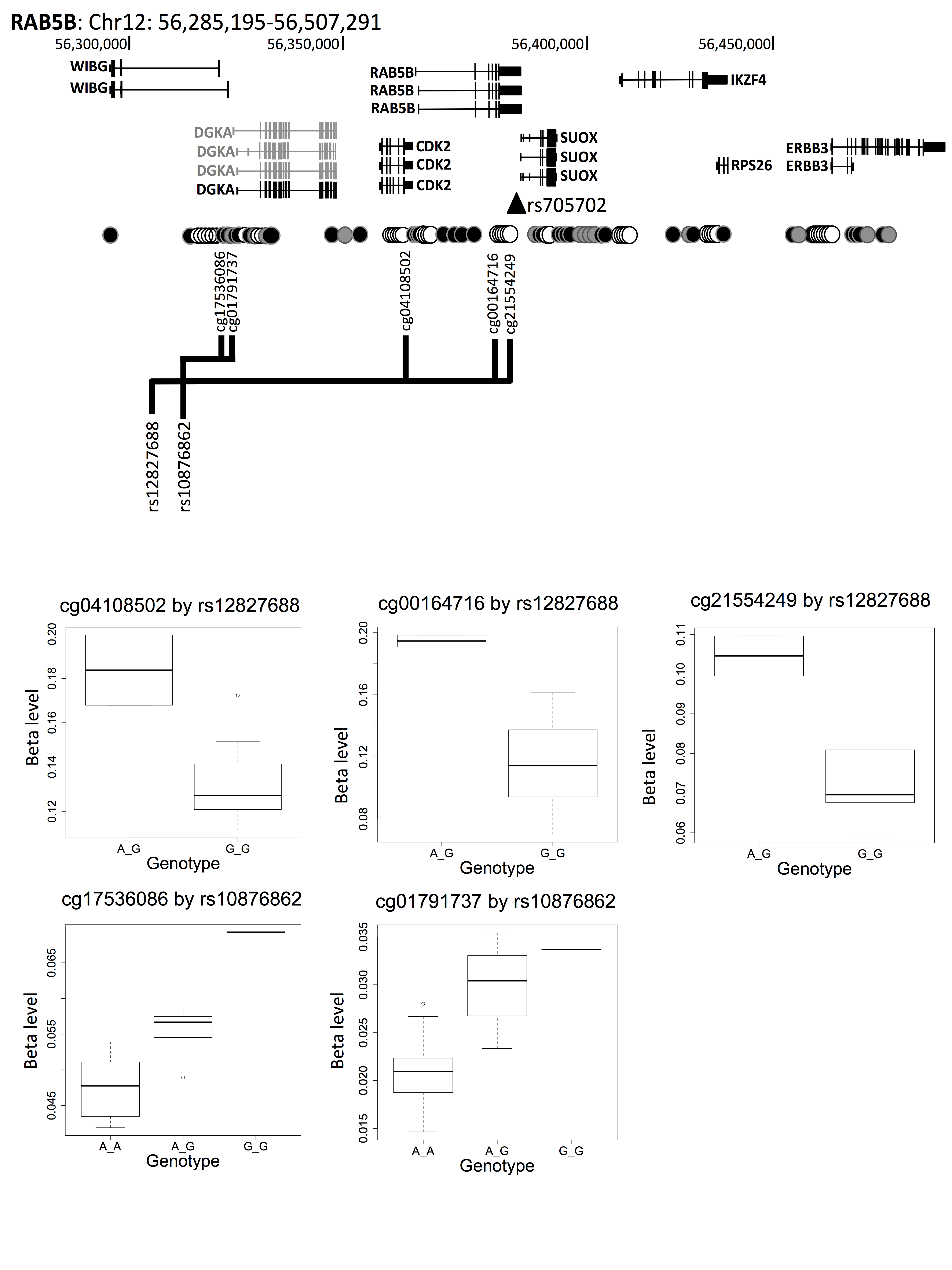

Supplement: S1 Fig — Genomic co-ordinates and gene position are shown in the top of the panel with the location of the PCOS GWAS index SNP shown as a solid triangle. Methylation status of each CpG residue is demonstrated by a circle (open = low-methylated, grey = semi-methylated, black = highly-methylated), with CpG-SNP interactions shown by a black line connecting associated methylation probes and SNPs. Box and whisker plots below show methylation level by genotype for each meQTL. (TIFF) [file pgen.1005455.s006.tiff]

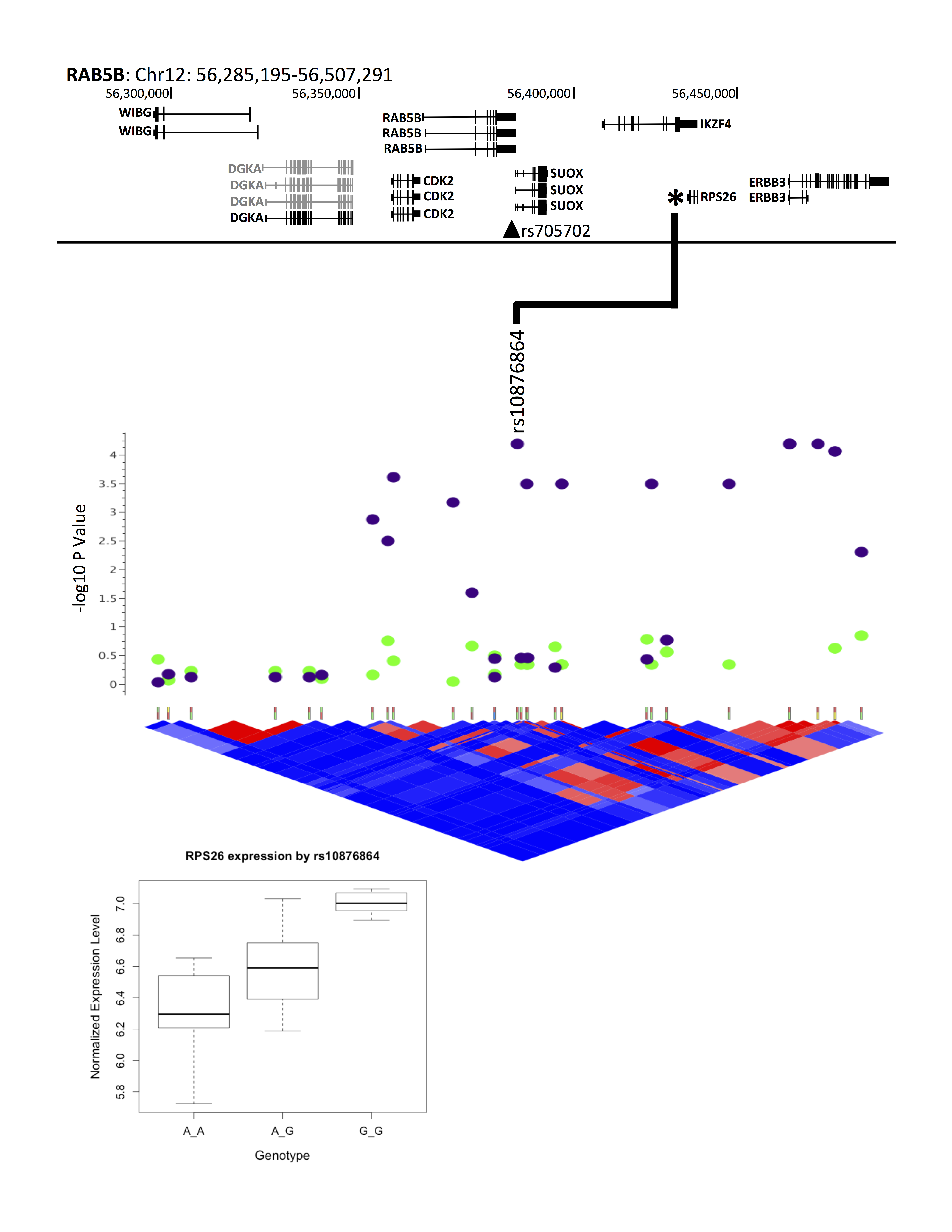

Supplement: S2 Fig — Genomic co-ordinates and gene position are shown in the top of the panel with the location of the PCOS GWAS index SNP shown as a solid triangle. Methylation status of each CpG residue is demonstrated by a circle (open = low-methylated, grey = semi-methylated, black = highly-methylated), with CpG-SNP interactions shown by a black line connecting associated methylation probes and SNPs. Box and whisker plots below show methylation level by genotype for each meQTL. (TIFF) [file pgen.1005455.s007.tiff]

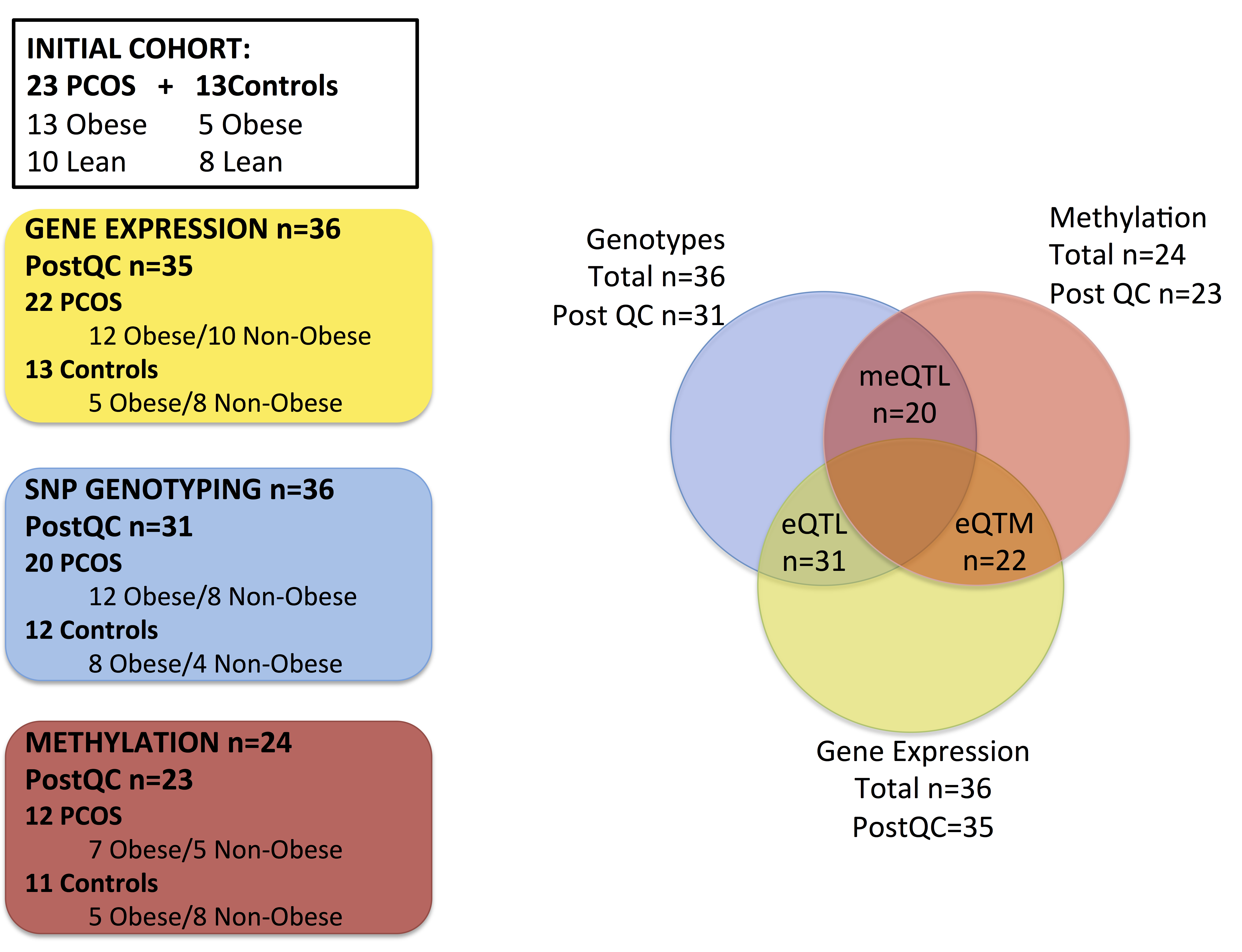

Supplement: S3 Fig — The samples used in this this study and the datasets generated for each. (TIF) [file pgen.1005455.s008.tif]
